# Auditory metabolomics, an approach to identify acute molecular effects of noise trauma

**DOI:** 10.1038/s41598-019-45385-8

**Published:** 2019-06-25

**Authors:** Lingchao Ji, Ho-Joon Lee, Guoqiang Wan, Guo-Peng Wang, Li Zhang, Peter Sajjakulnukit, Jochen Schacht, Costas A. Lyssiotis, Gabriel Corfas

**Affiliations:** 1 Kresge Hearing Research Institute and Department of Otolaryngology - Head and Neck Surgery, University of Michigan Medical School, 48109 Ann Arbor, USA; 2 Department of Molecular and Integrative Physiology, University of Michigan Medical School, 48109 Ann Arbor, USA

**Keywords:** Metabolomics, Cochlea

## Abstract

Animal-based studies have provided important insights into the structural and functional consequences of noise exposure on the cochlea. Yet, less is known about the molecular mechanisms by which noise induces cochlear damage, particularly at relatively low exposure levels. While there is ample evidence that noise exposure leads to changes in inner ear metabolism, the specific effects of noise exposure on the cochlear metabolome are poorly understood. In this study we applied liquid chromatography-coupled tandem mass spectrometry (LC-MS/MS)-based metabolomics to analyze the effects of noise on the mouse inner ear. Mice were exposed to noise that induces temporary threshold shifts, synaptopathy and permanent hidden hearing loss. Inner ears were harvested immediately after exposure and analyzed by targeted metabolomics for the relative abundance of 220 metabolites across the major metabolic pathways in central carbon metabolism. We identified 40 metabolites differentially affected by noise. Our approach detected novel noise-modulated metabolites and pathways, as well as some already linked to noise exposure or cochlear function such as neurotransmission and oxidative stress. Furthermore, it showed that metabolic effects of noise on the inner ear depend on the intensity and duration of exposure. Collectively, our results illustrate that metabolomics provides a powerful approach for the characterization of inner ear metabolites affected by auditory trauma. This type of information could lead to the identification of drug targets and novel therapies for noise-induced hearing loss.

## Introduction

Noise-induced hearing loss (NIHL) affects more than 300 million people worldwide, and 10% of the world’s population is exposed to potentially damaging sounds on a daily basis^1^, making noise exposure one of the most common causes of sensorineural hearing loss. Noise can affect the inner ear in two main ways, either by directly damaging tissues through the physical forces brought on by the sound waves, or by inducing molecular changes that then impact the health and function of inner ear cells or neurons. The severity of acoustic trauma and the resulting NIHL depends on the intensity and the duration of noise exposure. Loud sounds might cause a permanent threshold shift, i.e. overt hearing loss (OHL), which has traditionally been well investigated in patients and animals. In this case, a broad set of structures in the cochlea can be damaged, including stereocilia, hair cells, supporting cells and even the tectorial membrane^2^. Another type of NIHL that has received attention lately, hidden hearing loss (HHL)^3^, can occur upon exposure to moderate noise that only causes temporary shifts in hearing thresholds but permanently impairs sound-evoked neurotransmission, i.e. HHL is characterized by a decreased amplitude of the first peak of the auditory brain stem response (ABR) waveform that reflects the activation of the auditory nerve^4^. Noise-induced HHL is believed to be caused by the loss of synapses (synaptopathy) between inner hair cells and fibers of high-threshold spiral ganglion neuron^5^. Consequently, it has been suggested that HHL in humans leads to speech perception difficulties in noisy environments, while hearing thresholds remain normal^6,7^.

Despite the enormous impact of NIHL, the molecular mechanisms by which noise trauma damages the inner ear remain inadequately understood. The effects of noise are usually very rapid, e.g. synaptopathy is evident immediately after a two-hour exposure^8^, suggesting that they are mediated by changes in metabolism, which can occur similarly rapidly, as opposed to resulting from effects on gene expression, which can take hours to manifest. Yet, little is known about the effects of noise on the inner ear metabolome. Previous work on metabolic changes associated with NIHL has focused on OHL and on candidate molecules known to be involved in central nervous system trauma, e.g. glutamate, reactive oxygen species (ROS) and inflammatory mediators^9–14^. Unfortunately, pharmacological approaches targeting these pathways have not produced clinical treatments that effectively prevent noise-induced synaptopathy or hair cell loss, likely a reflection of the complexity of the biochemical changes induced. To better understand the effects of noise on the inner ear, we developed an auditory metabolomics pipeline that provides a comprehensive overview of the cochlear metabolome during noise exposure. Our results identified several metabolites and pathways influenced by noise, including some already linked to exposure or cochlear function, e.g. glutamate and NAD+, as well as numerous metabolites that have not been reported previously. This approach also shows that the effects of noise on the inner ear metabolome depend on the intensity and duration of the exposure. We believe that metabolomics is a powerful tool to define the ways in which the inner ear is affected by noise and will help identify the key molecular pathways that contribute to the different types of NIHL. This information would be helpful in guiding the design of new therapeutic approaches.

## Results

### Effects of an HHL-inducing noise exposure on the inner ear metabolome

**Figure 1:**
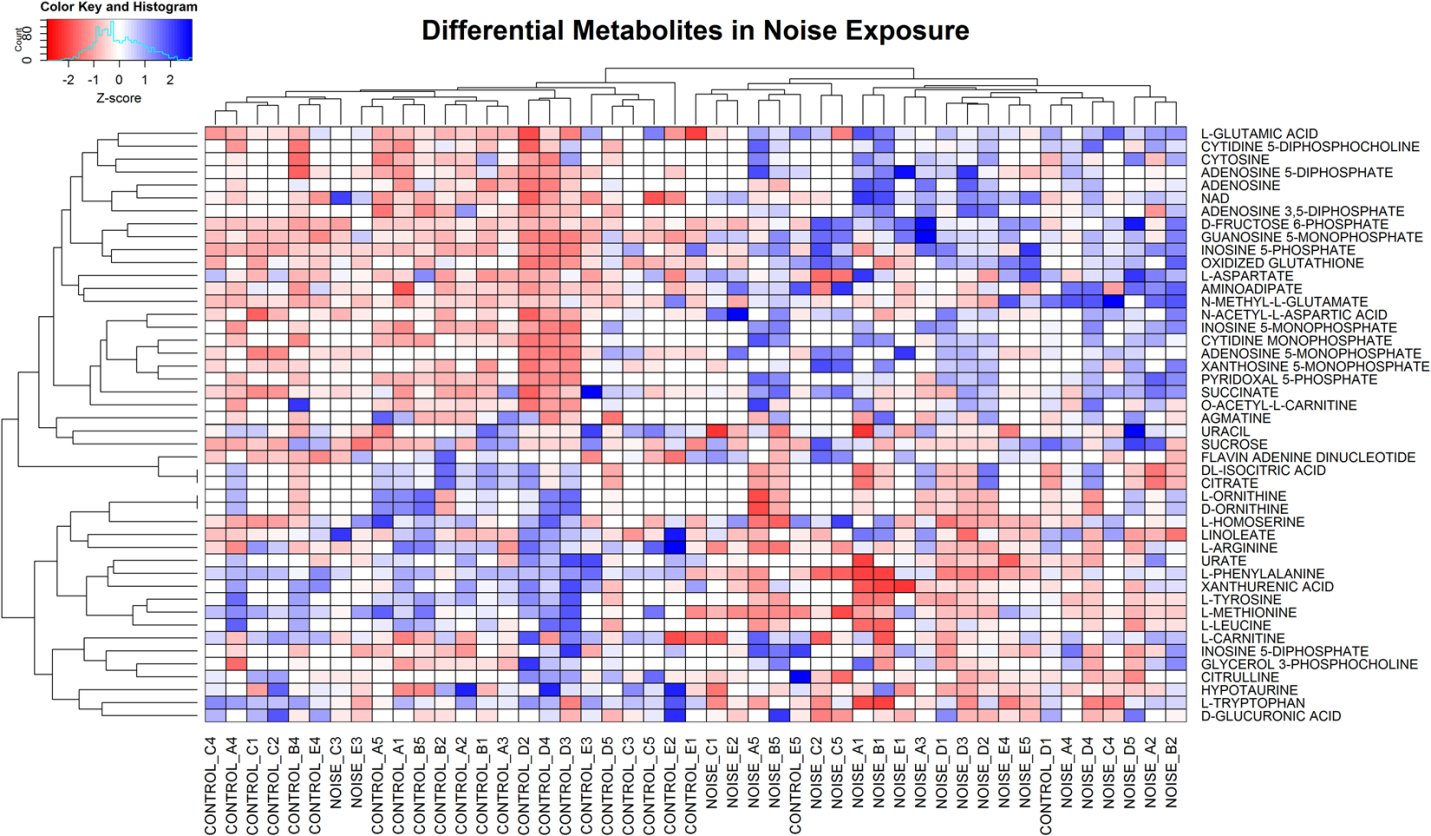
Differential metabolites and unsupervised hierarchical clustering. Data sets resulting from the LC-MS/MS runs of inner ears of control and noise-exposed mice (after filtering out under-loaded samples) were subjected to unsupervised hierarchical clustering. The control and noise-exposed samples segregate according to exposure with the exception of a few samples. The suffixes of “A1” to “E5” in the sample names represent 3 biological replicate experiments with each LC method and mice numbers: A = experiment 1 with RPLC, B = experiment 2 with RPLC, C = experiment 2 with HILIC, D = experiment 3 with RPLC, and E = experiment 3 with HILIC.

**Figure 2:**
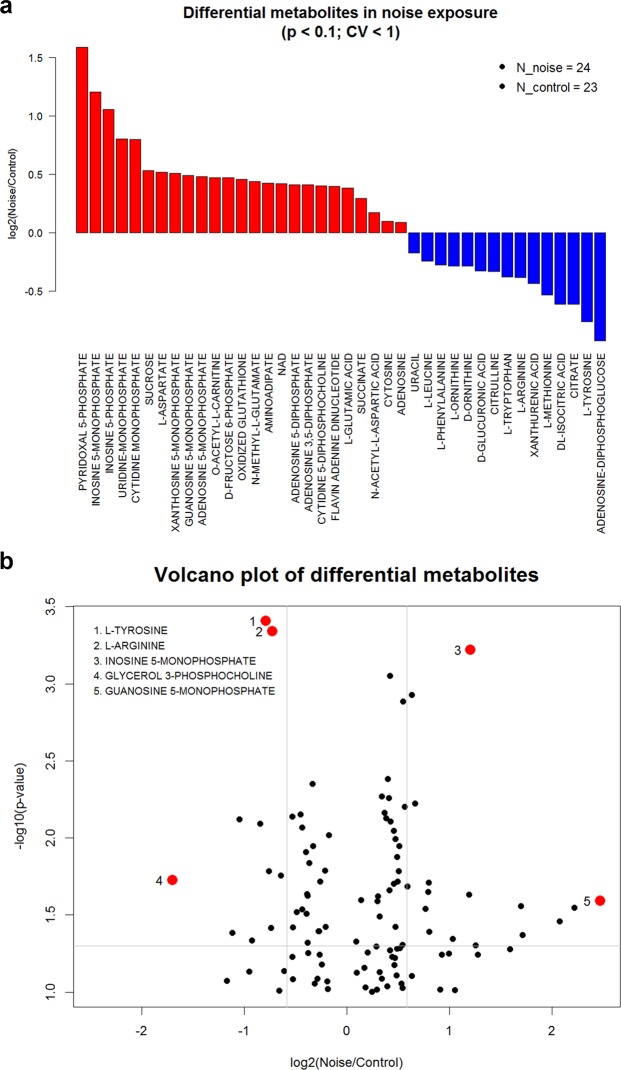
Metabolites altered by noise exposure. (**a**) Waterfall plot depicting the fold changes in the levels of metabolites affected by exposure to 100 dB SPL, 8–16 kHz for 2 hr. (**b**) Volcano plot of fold change vs. p-value. All differential metabolites are consistent in at least 2 experimental batches and the calculation of fold changes is based on median values of replicate measurements. See Supplementary Data for detailed values.

To begin exploring the effects of noise on the mouse inner ear metabolome, we exposed CBA/J mice to a noise band of 8–16 kHz at 100 decibels sound pressure level (dB SPL) for 2 hours, which induces temporary threshold shifts, synaptopathy and permanent hidden hearing loss^4,8^. We performed three biological replicate experiments, each with 5 animals per group (exposed and control). Temporal bones were harvested immediately after the exposure and the samples analyzed by liquid chromatography tandem mass spectrometry (LC-MS/MS). We measured the abundance of more than 220 metabolites which represent metabolic pathways in central carbon metabolism, including glycolysis, mitochondrial metabolism, amino acids, nucleotides, among others (see Methods section).We performed a two-tailed t-test to identify metabolites with significant differences between exposed and control samples in each experiment. Differential metabolites were defined as those with p-values < 0.1 and CV < 100% among replicates. This group of metabolites was considered for further analysis if they were consistent across the three experiments and present in at least 50% of all samples. These criteria identified 40 differential metabolites influenced by noise exposure. We then subjected this group of 40 metabolites to unsupervised hierarchical clustering (Fig. 1), which showed a good separation of control and noise-exposed samples. As shown in Fig. 2, this analysis identified 25 up-regulated and 15 down-regulated metabolites. The up-regulated metabolites include nucleotides, cofactors, and carbohydrates, as well as glutamate. Most of the down-regulated metabolites are amino acids such as methionine and arginine. Pathway analysis of the differential metabolites showed that up-regulation impacts most significantly alanine, aspartate, purine, glutamine and glutamate metabolism, while the down-regulated metabolites are involved in phenylalanine, tyrosine, and tryptophan metabolism (Table 1).

**Table 1:** Metabolic pathways altered by a 100 dB SPL, 8–16 kHz 2 hr noise exposure.

Pathway	P-value	FDR
**Alanine, aspartate and glutamate metabolism**	0.00045298	0.021229
**Purine metabolism**	0.00051779	0.021229
*Phenylalanine, tyrosine and tryptophan biosynthesis*	0.00046167	0.018928
*Phenylalanine metabolism*	0.0040809	0.11154

The top 2 up-regulated (bold) and down-regulated (italic) metabolic pathways identified by the MetaboAnalyst webtool. P-value is calculated by a hypergeometric test. FDR = false discovery rate.

### Metabolic changes depend on intensity and duration of noise exposure

**Figure 3:**
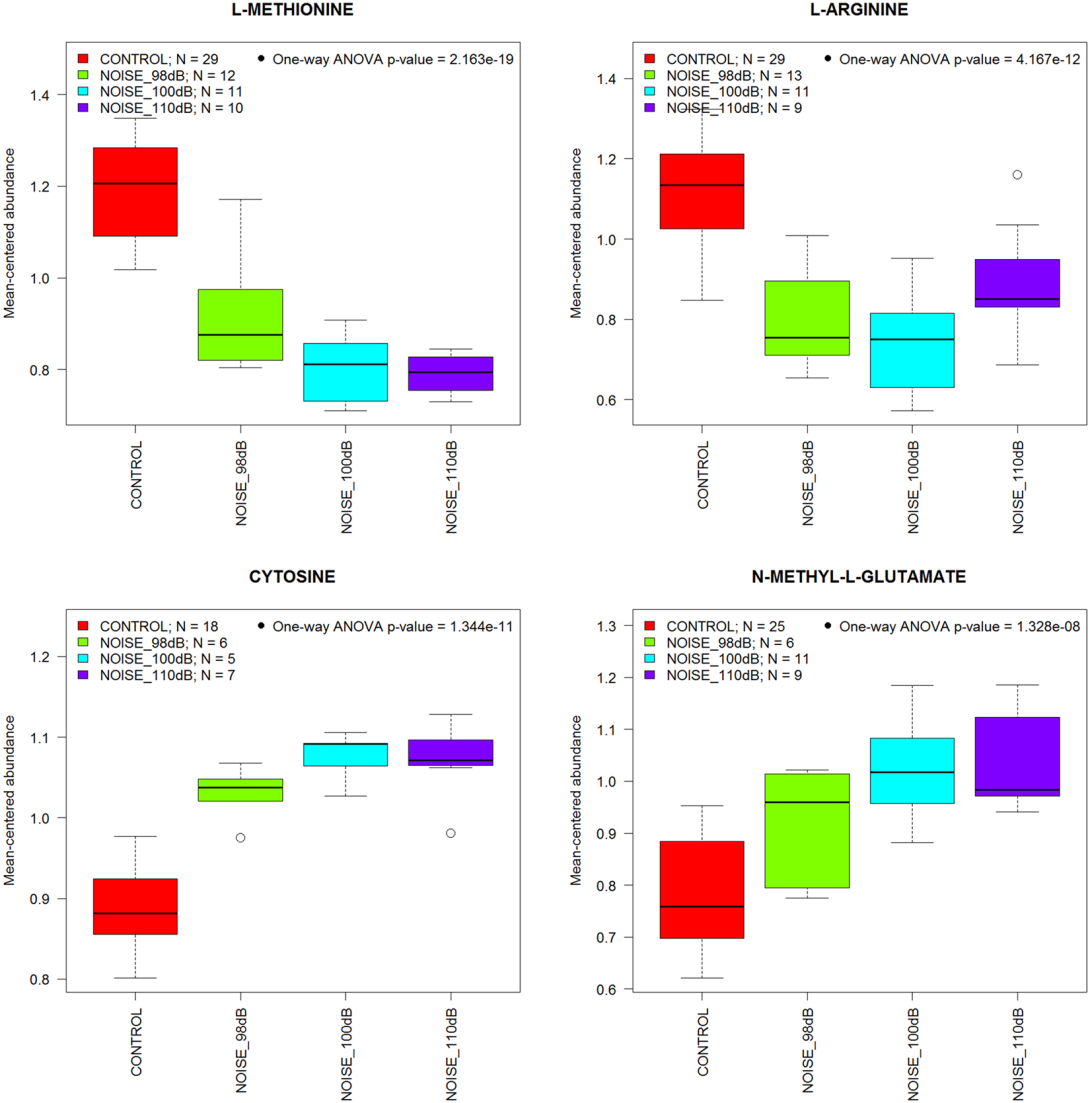
Intensity-dependent effects of noise on metabolite levels. The box-and-whisker plots illustrate that metabolic changes in the inner ear depend on the intensity of the sound presented (2 hours, 8–16 kHz band).

**Figure 4:**
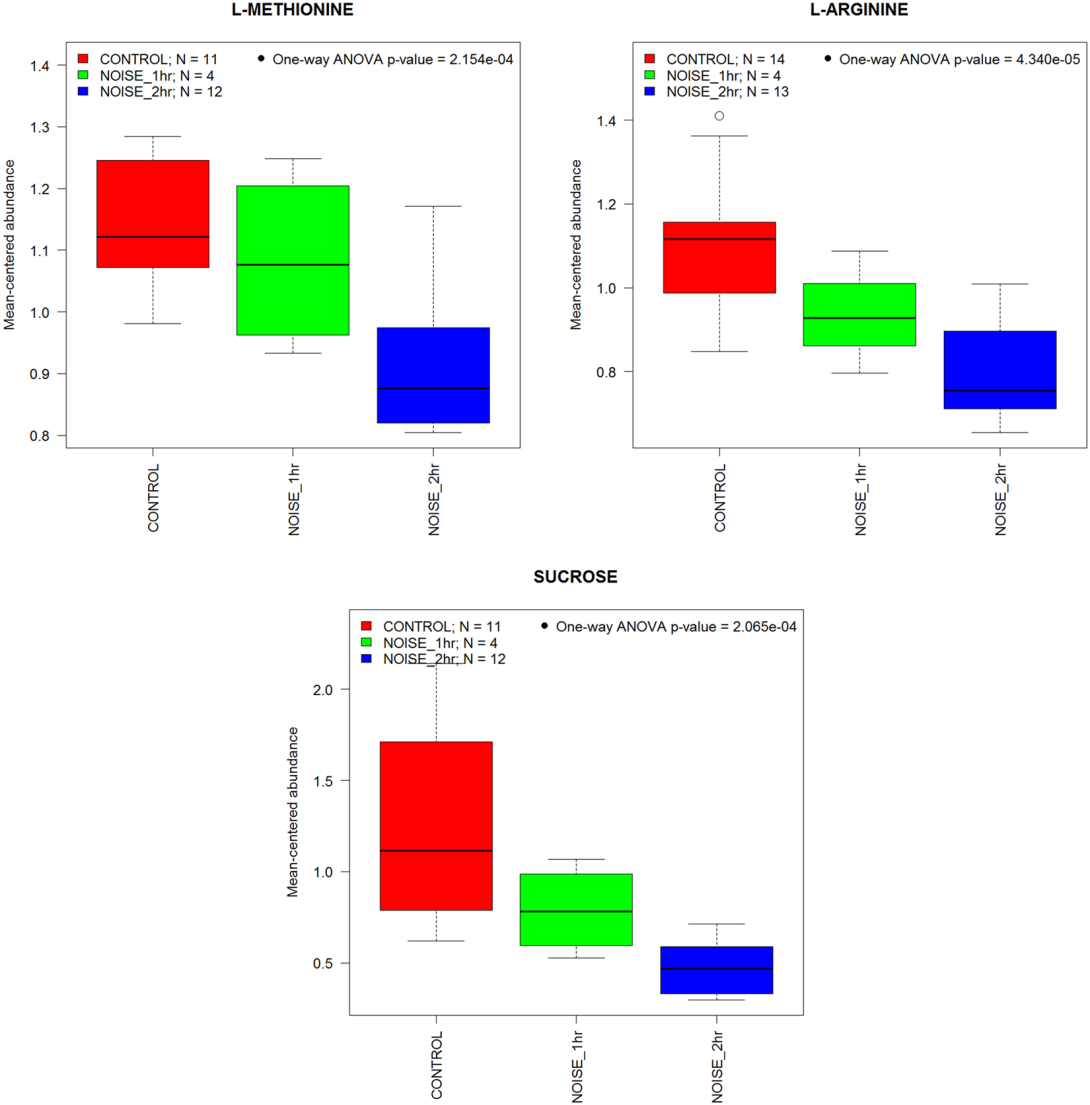
Duration-dependent effects of noise on metabolite levels. The box-and-whisker plots illustrate that metabolic changes in the inner ear depend on the duration of the exposure (98 dB SPL, 8–16 kHz band).

The well-documented pathological effects of noise on inner ear structure and function are intensity- and duration-dependent^2^. To test if the same is true for the effects of noise on the inner ear metabolome, we compared the effects of different types of exposures (Fig. 3). Representative results from four metabolites show a clear intensity-dependent effect (98, 100 or 110 dB SPL, 8–16 kHz, for 2 hr). As the noise intensity increases from 98 to 110 dB, so do the noise-induced changes in cytosine, N-methyl-L-glutamate, L-methionine and L-arginine. Similarly, representative results from three metabolites (Fig. 4) demonstrate that the effect of a 98 dB SPL, 8–16 kHz noise exposure depends on exposure duration (for 1 hr or 2 hr).

### Meta-analysis for robustness and statistical power

**Figure 5:**
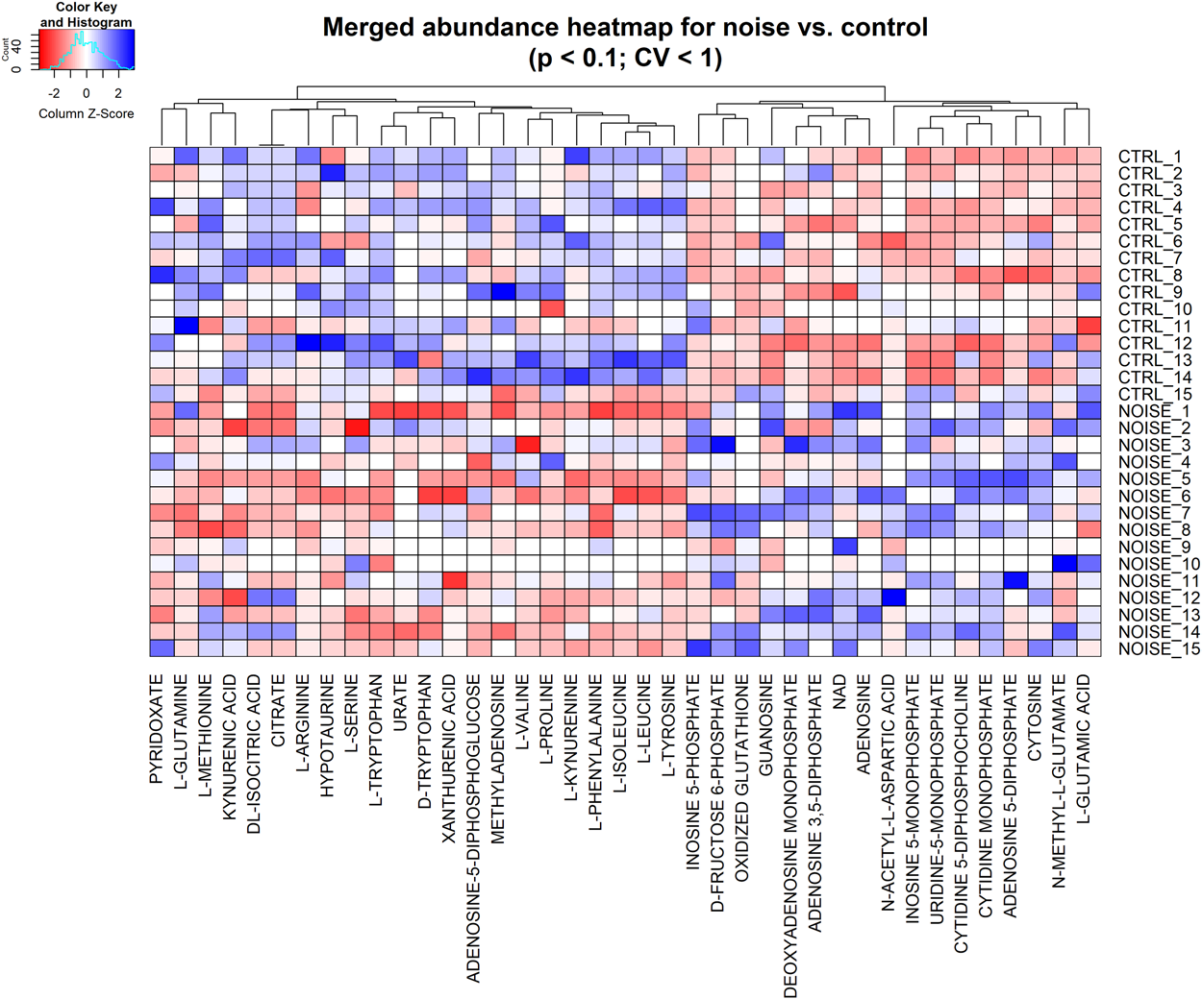
Meta-analysis of multiple datasets. Five metabolomics datasets from the three experiments with 100 dB SPL noise for 2 hours were analyzed by pooling all noise-exposed and control samples in two groups. Identification of differential metabolites and unsupervised hierarchical clustering were performed as in Fig. 1.

In order to assess the robustness of the above results with more statistical power, we performed an additional analysis of the samples with 100 dB SPL noise for two hours and the corresponding control samples by pooling all control and exposed samples into two groups after metabolite-wise data standardization of each dataset, thereby increasing the sample size in each group, i.e., 15 samples per condition. We analyzed a subset of 186 metabolites that have less than 5 missing values in each of the exposed and control groups and CV < 1 in all replicate groups across all datasets. Our bioinformatic analysis yielded 39 differential metabolites, of which 17 were up-regulated and 22 were down-regulated (Fig. 5). Twenty-three of those 39 differential metabolites were also found in our individual batch-based analyses above, which include glutamate, methionine, arginine, tryptophan, xanthurenic acid, NAD+, and oxidized glutathione (GSSG).

## Discussion

Our results show that metabolomics profiling is a powerful approach for the characterization of inner ear metabolites in normal and pathological states. We found that noise exposure induces consistent and significant metabolic changes on an acute time scale. The results of our targeted metabolomics approach on the relative abundance of 220 metabolites in freshly harvested mouse inner ears both validate the procedure and provide novel insights into noise trauma. The fact that compounds related to cochlear neurotransmission and metabolic stress are clearly altered by noise underscores the validity of the methodology, as does the fact that the metabolic changes induced by noise depended on the intensity and duration of the exposure. Importantly, bioinformatics analysis identified several pathways previously unknown to be altered by noise, indicating that this technology can provide novel insights into the molecular events triggered by noise trauma.

Only two studies on inner ear metabolomics have been published previously. Fujita *et al*.^15^ measured the effects of a strong noise exposure (126 dB SPL) on the metabolome of guinea pig inner ear fluids while Mavel *et al*.^16^ analyzed the perilymph of humans who were undergoing cochlear implantation. We chose to analyze the whole mouse inner ear since at these early stages of technique development and exploration we believed it is imperative to consider the effects of noise broadly on metabolites in both intracellular and extracellular inner ear compartments. An added power of our approach is that future use of mouse models will provide tools to explore the relationship between a specific cell type (e.g., hair cells) or function (e.g., mechanotransduction) and the effects of pathological conditions on inner ear metabolism.

Our results on the effects of noise differ significantly from those of Fujita *et al*.^15^. Specifically, we measured the abundance of 220 metabolites, while their approach allowed for the detection of only 77. Consequently, we detected a larger number of noise-induced changes in inner ear metabolites, i.e. we identified 40 differential metabolites in whole inner ears while they only found 10 in inner ear fluids. Furthermore, more than half of our differential metabolites were undetectable in their experiment, and only 28% of metabolites altered by noise in our samples were detectable in theirs. The most parsimonious explanation is that the differences are due to the contents of inner ear tissues that are included in our samples although methodological disparities might have contributed. Importantly, in contrast to our analysis of the whole cochlea, Fujita *et al*.^16^ did not find effects of noise on neurotransmitters and compounds indicative of oxidative stress in guinea pig inner ear fluids, indicating that analysis of the cell compartments is critical.

Metabolomics has been also used to study the effects of noise exposure on the brain^17^. Interestingly, this study showed that a 1 h exposure to 16 kHz 110 dB SPL can cause long-lasting changes in the brain metabolome 6 months post-exposure. While the metabolic changes we observed in the inner ear are a direct effect of the sound, it is likely that the changes in the brain are a consequence of the noise-induced hearing loss.

The 220 metabolites we selected for analysis encompass the major pathways in central carbon metabolism and can thus be hypothesized as early indicators of pathological changes. Table 1 highlights the four pathways that showed the strongest statistical significance, as determined by pathway analysis for the top 40 altered metabolites identified using the MetaboAnalyst tool (http://www.metaboanalyst.ca/). Glutamate is the principal afferent neurotransmitter in the auditory system, mediating synaptic transmission between inner hair cells and afferent spiral ganglion neurons. Excess glutamate release and hyperactivation of glutamate receptors in the cochlea have been linked to excitotoxicity and damage to hair cells’ afferent terminals^18–20^. In particular, glutamate excitotoxicity has been suggested to contribute to the synaptic swelling seen after intense acoustic trauma (130 dB SPL)^11,21,22^. However, previous studies failed to show a glutamate involvement during less intense exposures (100 dB or 105 dB SPL)^21,23^. The up-regulation of glutamate in response to moderate noise (100 dB SPL) seen in our study extends the notion of glutamate excitotoxicity contributing to the pathogenesis of noise-induced HHL and synaptopathy. Xanthurenic acid (XA), an inhibitor of the glutamate vesicular transporter VGLUT^24^, is down-regulated in our samples, suggesting that VGLUT activity might increase after noise. In addition to glutamate, aspartate is a major excitatory transmitter in the brain that is enriched in hair cells, afferent and efferent nerve fibers, and supporting cells in inner ear^25^. Our observed increase after noise is consistent with Jaeger *et al*. and might be larger than or at least equal to the response of glutamate^23^. Aspartate, therefore, could play a role similar to glutamate in inner ear metabolism and noise-induced hearing loss.

The indication that noise reduces phenylalanine, tyrosine and tryptophan biosynthesis is intriguing because a previous study showed that these aromatic amino acids suppress discharge by afferent fibers innervating hair cells in the lateral line of Xenopus^26^. Thus, our data raise the question whether alterations in these amino acids are also at play in the mammalian cochlea and if they might contribute to vulnerability to noise.

It is likely that some of the noise-induced metabolic changes we observed reflect endogenous mechanisms of oto-protection. This might be the case for the noise-induced increases in ADP, AMP and adenosine levels (Fig. 2) as purinergic signaling has been suggested to protect from noise trauma and contribute to cochlear adaptation to elevated sound levels^27,28^. Intense acoustic-overstimulation can increase inner ear purinergic signaling by enhancing agonist levels^29^, increasing receptor levels^30^, and upregulating the enzyme that catalyzes the breakdown of purinergic ligands, NTPDase^31,32^. Adenosine signaling via the A1 receptor, which was shown to be partially upregulated by noise in cochlear tissues^33^, has also been linked to protection from noise-induced cochlear injury through the activation of antioxidant enzymes, inhibiting the release of neurotransmitters and promoting anti-apoptotic pathways^34–37^.

The emergence of oxidative stress and its counterbalance by the action of antioxidant systems, well documented in noise trauma^38^, also is reflected in our results. The increase in the oxidized form of the electron carriers NAD^+^ and FAD^+^ indicates a drain on reducing power which is underscored by an increase in oxidized glutathione, the major cellular antioxidant. Methionine, which is depleted following noise, can be recruited for the synthesis of glutathione or the provision of sulfhydryl groups via cysteine. The observation of decreased methionine also provides an explanation for the efficacy of supplementary methionine to attenuate noise- and drug-induced hearing loss in animal models^38–40^.

In summary, we show that MS-based metabolomics is a powerful tool to study global metabolic effects induced by noise on the inner ear in mice. Noise exposures that result in hearing loss, hidden or overt, trigger acute metabolite abundance changes. It remains a challenge to answer the outstanding questions of which cell types give rise to the metabolic signatures and which of the changes are most significant and causal to the ensuing pathology. Towards this goal, we were able to identify differential inner ear metabolites and metabolic pathways that were significantly affected by noise depending on exposure intensity and duration. Most notably, our data have added to the understanding of the complex neurotransmission and modulation pathways altered by noise, and further supports the involvement of oxidative stress in noise trauma. This study therefore provides insights into the pathophysiology of NIHL from a metabolic perspective and offers novel opportunities to study pathogenesis, biomarker discovery, and to identify therapeutic strategies to block or treat disease.

## Methods

### Experimental groups and noise exposure

All animal procedures were approved by Institutional Animal Care and Use Committee of University of Michigan and all experiments were performed in accordance with relevant guidelines and regulations. For this study we used CBA/J mice with normal hearing housed in a facility with ambient sound levels of 40–50 dB within frequencies detectable by the mouse ear. Animals (8–12 weeks old) were randomly assigned to a control or noise exposed group (see Table 2). Control groups (n = 45) received no noise exposure. awake mice were placed within small cells in a subdivided cage, suspended in a reverberant noise exposure chamber. The stimulus was an exposure to an octave-band noise (8–16 kHz). Noise levels in different groups varied from 98 to 110 dB SPL and noise duration varied from 1 hour to 2 hours (Table 2). Noise calibration to target SPL was performed before each exposure session. Sound pressure levels varied by <1 dB across the cages.

**Table 2:** Noise exposure intensity and duration used in this study.

Intensity	Duration	Number of mice
(dB)	(hour)	
100	2	15
98	2	15
98	1	5
110	2	10

### Sample preparation

Temporal bones were harvested immediately after the exposure and the auditory bulla were isolated from the surrounding skull bone and placed in a Petri dish with phosphate buffered saline (PBS). We then quickly removed any attached musculature or soft tissue, the middle ear and the cerebellar parafloculli to isolate the bony otic capsule containing the entire cochlea and vestibular organs (including sensory epithelia, sensory neurons, nerves, etc.) for processing. The round and oval windows were kept intact during the dissection to prevent dilution or contamination of the inner ear fluid with the bath solution.

Both inner ear tissues from a single mouse were placed together in an individual tube containing 1 ml of 80% methanol in water at −80 °C and stored at the same temperature. Samples of one batch (five exposed and five unexposed mice) were then simultaneously homogenized in their individual tubes using steel beads and a Qiagen Tissue Lyser (45 seconds at 28 Hz at room temperature). Samples were immediately brought back to −80 °C, after which insoluble material was centrifuged at 14,000 g at 4 °C. Supernatants were transferred to new tubes and subjected to Speedvac evaporation to obtain a dry pellet for liquid chromatography-coupled tandem mass spectrometry (LC-MS/MS) analysis.

### Targeted metabolomics analysis

Metabolomics analysis was done using in-house protocols^41,42^. Briefly, samples were analyzed using an Agilent 1290 UHPLC and 6490 Triple Quadrupole (QqQ) Mass Spectrometer (LC-MS/MS). We employed two LC methods: reversed-phase liquid chromatography (RPLC) and hydrophilic interaction liquid chromatography (HILIC). The RPLC separates hydrophobic or non-polar metabolites, while the HILIC separates polar or ionic metabolites. This approach generated two data sets per batch resulting in relative quantification ~220 metabolites across the major metabolic pathways in central carbon metabolism, including glycolysis, the TCA cycle, pentose phosphate pathway, amino acid and nucleotide metabolism, among others^42^.

### Bioinformatic and statistical analysis

The pre-processed data of LC-MS/MS raw signals were post-processed to facilitate meta-analysis of differential metabolites from multiple datasets. We have developed a bioinformatic analysis pipeline in the R language based on our previous work^43^. We first calculated coefficient of variation (CV) across replicate samples for each metabolite given a cut-off value of signal peak areas in each dataset. We visually inspected distributions of CVs across multiple peak-area thresholds to identify the best threshold in each dataset as a noise cut-off value. Each sample was then normalized by the total intensity of all metabolites to correct for the moderate differences among tissue samples. Each metabolite abundance profile was scaled by the mean abundance across all samples for statistical analyses and visualizations. We performed a two-tailed t-test to identify metabolites with significant differences between exposed and control samples in each experiment. Differential metabolites were defined as those with p-values < 0.1 and CV < 1 among replicates. Metabolites were considered for further analysis if their profiles were consistent across the three experiments, and if present in at least half of all samples. Pathway analysis was performed using the MetaboAnalyst webtool^44^.

## Supplementary information

Supplementary data

All data sets
