# Supplementary material for: Auditory metabolomics, an approach to identify acute molecular effects of noise trauma
Source: Sci Rep. 2019 Jun 25;9:9273. doi: 10.1038/s41598-019-45385-8 (PMC6592947; doi:10.1038/s41598-019-45385-8)
Supplement: Supplementary file 1 — Supplementary data [file 41598_2019_45385_MOESM1_ESM.docx]

**Auditory metabolomics, an approach to identify acute molecular effects of noise trauma**

**Lingchao Ji^1^*, Ho Joon Lee^2^*, Guoqiang Wan^1^, Guo-Peng Wang^1^, Li Zhang^2^, Peter Sajjakulnukit^2^, Jochen Schacht^1^, Costas A. Lyssiotis^2, #^ and Gabriel Corfas^1, #^**

**Supplementary data**

The Supplementary data file contains all data associated with this, including the processed data for Figures 1 – 5 and all raw data.
